# 

**Published:** 1888-02-11

**Authors:** 


### The Students' Column.

### ROYAL COLLEGES OF PHYSICIANS AND SURGEONS, EDINBURGH, AND FACULTY OF PHYSICIANS AND SURGEONS, GLASGOW.

### TRIPLE QUALIFICATION.

The Quarterly Examinations in Edinburgh for the Triple Qualification took place in January, with the following results:—

### FIRST EXAMINATION.

Of 58 candidates the following 37 passed: Patrick Francis O'Hagan, Longford; George Stevens Pope, Madras; Gordon Trafforde Tuke, Edinburgh; Arthur Henry Barstow, Spofforth; John Francis Roden, Edinburgh; George Arthur Ings, Canada; Ernest Victor Eames, Donegal; Thomas Jobling Tonkin, London; Thomas Sprot Allan, Glasgow; James Stevenson, Dundee; William Henry Walker, Ripon; Francis Joseph Flavin, Doncaster; James Francis Curry, Limerick; Thomas Henry Hosford, co. Cork; Richard Rainey Workman, co. Derry; Herman Stedman, London; Hugh Shaw, Enniscorthy; Harry Albert Holmes, Manchester; Patrick Regan Crofton, Ireland; Charles Joseph Adams Coates, co. Cork; William Yeates, co. Down; Richard Snaith Jaques, Scarborough; Archibald Robertson Douglas, Newcastle-on-Tyne; David Cameron Carnduff, Berhampore; Robert Milling, co. Down; George Gunn Sinclair, Hamilton; William Wendt Margenout, Ceylon; George Maingot, Trinidad; Robert Love, co. Antrim; Albert Burns, Chatham; Miss Beatrice Mary Harrison, Brighton; Charles Edward Dodd, Cheshire; Francis Cubbon Rogers, Cheshire; Newton John Newbould, Cambridge; Joshua Hamilton Hart, Yorkshire; Augustus Henry Dubourg, London; and Charles Edward Gooderson Bateman, Norwich.

### SECOND EXAMINATION.

Of 63 candidates the following 34 passed:—John Round, Dudley; John Paterson McLaren, Glasgow; Robert Aldous, Norfolk; Charles James Milligan, Belfast; Thomas Simpson Hogg, co. Derry; Samuel William Wolfe, co. Cork; Cecil Lucius Strangman, Waterford; Charles Edward Dodd, Minshul Vernon; Newton John Newbould, Cambridge; Francis Cubbon Rogers, Cheshire; William Cooke Lancaster, Dublin; Edward Treharne, Glamorganshire; Robert Whiteside Morrow, co. Down; John Birtwhistle Griffiths, Stroud; John Henry Carson, co. Down; William Wendt Margenout, Ceylon; Edmund Raymond Carroll, co. Cork; Joseph Ledger Smith, co. Limerick; Thomas Stephen MacMahon, Longford; Tom Balleny Brooke, Cambridgeshire; John MacKenzie, Sutherland; Herbert Edmund Wright, Oldham; James Stobo, Bothwell; John Christopher Thompson, Yorkshire; John Quigley, Londonderry; James O'Sullivan, Dublin; Adam Ramage, Kilmarnock; Alfred Millar Ford, Glasgow; Thomas Frank Southam, Cheshire; Robert Johnstone Stirling, Peebles; James Maher, Waterford; William Smyth Crawford, co. Down; John Baldwin Meredith, Queen's co.; and Augustus Henry Dubourg, London.

### FINAL EXAMINATION.

Of 86 candidates the following 40 passed, and were admitted L.R.C.P.E., L.R.C.S.E., and L.F.P. & S.G.: Antonio Aluisio Jervis-Pereira, India; John Hoyle, Ashby-de-la-Zouch; Miss Mary Crawley, Northampton; Samuel Evans, Madras; William Augustus Gibson, Dublin; Richard Laurence Caunter, Cornwall; Arthur Joseph Ryan, co. Limerick; Maurice John O'Connell, Cork; Kilcoursie Jocelyn Courtenay, Sheerness; Edward Brooks, Blackburn; William James France, Shrewsbury; John Henry Lloyde, Aberystwyth; James Muir Crawford, India; John Conrad Scotchburn, Yorkshire; Charles Mattei, Malta; John Charles Palmer, Melbourne; Thomas Simpson Hogg, co. Derry; James Stoddart, Dumfriesshire; James Campbell MacDiarmid, Argyleshire; Edward Macready Spencer, Tavistock; John Cotter, Cork; Joseph McDowell, Newry; Michael McLaughlin, co. Donegal; Joseph Collin Heynsbergh, Ceylon; Morton Edmund Leicester, London; Robert King Mitter, India; Alfred William Douglas, Aldershot; Michael Thomas Casey, co. Limerick; Alfred Seymour Taylor, Walker-on-Tyne; Benjamin John Edward Wright, London; John Charles French, co. Durham; George Ernest Rawlinson, Oxford; John Timothy Kennedy, Kerry; Charles Doherty, co. Donegal; John Potterton Ferguson, Ireland; William Kerr Lochhead, New South Wales; James William Kelly, Queenstown; Clarence Beesley, Southsea; Thomas Ireland, Germany; and George Franklin Hilliard, Ireland.

### ROYAL COLLEGE OF SURGEONS, EDINBURGH.

During the January sittings of the Examiners, Mr. William George Sym, Edinburgh, passed the Final Examination, and was admitted L.R.C.S.E. For the Licence in Dental Surgery, Mr. Arthur Turner, Aylesbury, passed the First Professional Examination; and the following gentlemen passed the Final Examination, and were admitted L.D.S. Edinburgh:—Edward Arthur White, Stoke Holly Cross, Norwich; and John Turner, Edinburgh.

Questions in Elementary Anatomy (all of which are to be answered).—I. Enumerate the Foramina which exist in the sphenoid bone, and name the structures transmitted by them. II. Describe the Cutaneous Nerve supply of the toes. III. Give the course, relations, and branches of the Anterior Interosseous Artery. IV. Give the origin insertion, and nerve supply of the following muscles: Gluteus maximus, Tibialis anticus, and Supinator brevis.

Questions in Chemistry (four questions, all of which are to be answered).—I. Write the formulæ of the following substances: Lead acetate, mercuric chloride, black oxide of manganese, magnesia, peroxide of hydrogen. II. Explain the term "specific gravity" as regards solids and liquids. A sp. gr. bottle, holding 50 grams. of water, was found to hold 50·96 grams. of urine: calculate the sp. gr. of the urine. III. How is "yellow prussiate of potash" manufactured? Give its chemical name and formula. What takes place when chlorine gas is passed into a solution of the salt? IV. What takes place when the following re-agents are added to separate portions of a solution of a "lead" salt—$HCl$, $H_2S$, $KI$, $H_2SO_4$?

Questions in Advanced Anatomy (all of which are to be answered).—I. Describe the Lumbar Plexus, and name the branches derived from each of the nerves. II. Name the Cartilages of the Larynx, and describe the largest of these, indicating the points to which muscles and ligaments are attached. III Give the course and describe the branches of the first part of the Internal Maxillary Artery. IV. Specify the nerve supply of the following muscles: Levator labii superioris, Levator palpebræ, Digastricus, Sternomastoid, Rectus abdominis, Rectus externus, Rectus femoris, Stylo-glossus, Stylo-pharyngeus, Thyro-hyoid, Sterno-thyroid, and Lingualis.

Questions in Physiology (all of which are to be answered).—I. Give an account of the nerve mechanism of the heart—intrinsic and extrinsic. II. Describe the manner by which the "arterial tension" of the Carotid Artery has been estimated. III. What are the chief layers of the Blastoderm? and mention the most important structures and organs formed from each. IV. How is animal heat produced? what are its normal limits in man? and how is it maintained within these limits?

Questions in Materia Medica (three questions, all of which are to be answered).—I. What part of the Hyoscyamus plant is used in medicine. Mention the order to which it belongs, and the various preparations and doses. II. Mention the preparations and doses of magnesia. III State the composition of the Liquor hydrargyri perchloridi, and its dose.

Questions in Medicine (three only of which are to be answered).—I. Give the etiology, symptoms, pathology, and treatment of Exophthalmic Goitre. II. Mention four diseases of which polyuria is a symptom, and give the differential diagnosis of these from one another. III. Enumerate the symptoms and signs which might be present in a case of aneurism situated at the junction of the arch of the aorta with the descending aorta. Explain the treatment of such a case. IV. Give the etiology, symptoms, pathology, and treatment of Asiatic cholera.

Questions in Therapeutics (all of which are to be answered, including the prescription).—I. How may Hæmatemesis be most effectively treated; Explain the rationale of the treatment selected. II. Sketch a dietary to be used by a person of pronounced gouty constitution. III. Give the appropriate treatment for three forms of Diarrhœa. Prescription (to be written in unabbreviated Latin, the directions in English): Prescribe a draught containing bromide of potash, hyoscyamus, and chloroform water.

Questions in Surgery (all of which are to be answered).—I. Osteoma—(1) What varieties present themselves? (2) Describe the exostoses of the great toe, and of the skull. (3) What treatment would you adopt for each? II. Distinguish (1) pathologically, and (2) symptomatically, Chronic Rheumatoid Arthritis, Gonorrhœal Rheumatism, and Charcot's Ataxic Arthritis. III. Hydrops of the knee—Give (1) its causes; (2) its symptoms; (3) its treatment. IV. State (1) the varieties of hare lip, (2) the period for operating, (3) the mode of operating.—Surgical Anatomy (one of which only is to be answered): I. Describe the anatomy of the Parotid Gland, with special reference to excision of parotid tumours and the risks of that operation. II. Explain the arterial and venous distribution to the Rectum, and the arrangement of Sphincters and Levator Ani.

Questions in Medical Jurisprudence and Hygiene (four questions, of which three are to be answered; in these 3 and 4 must be included). I. Mention the Signs of Death. Describe the characters of the early stages of Putrefaction; and state the period of its oncoming in various parts of a body exposed in a mortuary at this time of year. II. Describe the differences produced by the application of heat to the body, before and after death. III. What are the more common poisonous Salts of Copper? How may Copper accidentally gain access to food or drink? What are the symptoms produced in acute poisoning therefrom, and how may the poison be detected in the contents of the stomach? IV. Describe the process of Disinfecting the room, bedding, &c., of a person who had lain in it ill of Scarlet Fever, giving your reasons for the various steps taken.

### SPECIAL NOTICE TO NEWSAGENTS, ADVERTISERS, AND THE TRADE.

### CHANGE OF OFFICE AND PUBLISHER.

Notice is hereby given, that the Office of The Hospital will henceforth be at 38, Old Jewry (Cheapside end), E.C., to which address all communications should be sent. Mr. A. Doig has been appointed Advertising Agent, and Mr. J. H. S. Hanning, Manager and Publisher. All Cheques and Post Office Orders should be made payable to J. H. S. Hanning, 38, Old Jewry, E.C., crossed Lloyds, Barnetts, and Bosanquets Bank (Limired).

### QUESTIONS AND ANSWERS.

WITH reference to Questions and Answers, "Hospitals for Incurables," (page 325 of THE HOSPITAL for 4th inst.), the Longmore Hospital, Edinburgh, admits patients free by the selection of the committee, and persons in receipt of parochial assistance by arrangement with the parochial authorities, on payment of a small weekly board. We have also private rooms for patients who can pay board—£52 for a whole room; £35 for a half-room.

J. T. MACLAGAN.
6, North St. David Street, Edinburgh.

### Amusements with Profit for all Ages.

**Rules.**—All answers must be addressed to the Prize Editor, The Hospital, 38, Old Jewry, Cheapside, London, E.C., not later than the *first* post on Thursday next. The full name and address must in all cases be given, but a *nom de plume* may be added for publication. Only one side of the paper must be written on.

### FOR MEN AND WOMEN.

### Acrostic Competition.

Commenced November 26th, 1887; ends February 18th, 1888.

A PRIZE OF ONE GUINEA will be given to the competitor who gains the highest number of marks for best original acrostics during the ensuing quarter. All acrostics sent in for this competition will be credited according to merit, twelve marks being the highest score given for one acrostic.

A PRIZE of HALF-A-GUINEA to be given to the competitor who gains the highest score for solution of acrostics set, including the one inserted on November 26th. One mark only will be given to the competitors who solve all the lights of each acrostic.

These competitions to alternate weekly.

Credit will be given this week for

### No. 6 Original Double Acrostic.

Results of No. 5 Original Double Acrostic.—Pasteur (12), Brownie (12), Mater (10), Ellice (9), E. M. B. (9), A. M. E. (9), Gretta (8).

### II. Word and Literary Competition.

### Results of First Quarterly Word and Literary Competition.

First Prize.—*Reldas* (4,389)—Miss A. Sadler, 32, Noiland Square.

Second Prize.—*E.E.* (4,324)—Miss Peach, 29, Comeragh Road, West Kensington, S.W.

Brisbane was very nearly successful, as she gained 4,314 marks, and will therefore be able to look forward with confidence to a prize if she perseveres, as we hope she may.

Literary Prize, 10s. 6d — *Gretta* — Miss Margaretta Parker, Effingham Villa, Park Lane, Stoke Newington.

Second Quarterly Competition commenced February 4th, 1888; ends April 28th, 1888.

Three prizes of one guinea, 15*s*. and 10*s*. 6*d*. will be given each quarter to the three competitors who obtain the highest number of marks: (1) by making the largest number of words derived from the word or phrase set for anatomical dissection; or (2) for the best answers to (6); or (3) by (1) and (2) combined.

(A) THE WORD COMPETITION.

We shall give the newspapers and periodicals for dissection during this quarter, that for this, the *second* week of the quarter, being

"Observer"

Observe.—Proper names, abbreviations, foreign words, words of less than four letters and repetitions are barred. Nuttall's *Standard* dictionary only to be used.

### Results of Thirteenth Week.

*Spectator.*—Ellice (346), Gretta (322), E. E. (283), Reldas (242), Lauriston (204), K. M. (200), Brisbane (196), Condorcet (194), Tabs (179).

(B) THE LITERARY COMPETITION.

Some people hate puzzles of all kinds, including word competitions, but they are fond of correspondence, and many sisters and mothers are continually receiving and sending letters to and from members of the family who are abroad India or the Colonies. The constant coming and going between England and other portions of the British Empire make it a matter of interest and importance to know something about the life, the climate, the amusements, the adventures, the clothing, and the surroundings of those who seek a livelihood beyond the seas. We shall therefore try to find space for the publication of selected letters from abroad, which may be sent by any of our readers who have friends and relations in foreign lands. We shall give marks for these letters as well as for the word competitions which will count equally for these prizes, so that anyone may enter for both or either, the prizes to be given to those who get the highest number of marks.

### Letters from across the Seas.

*Letter from Calcutta.*

From Calcutta to Benares.

I decided on my making my first stop at Benares, "The Holy City of the Hindoos." I sent my "baboo" out to look for business while I stayed at the hotel. On his return he said some might be done in a day or two, so resolved to go on to Lucknow and call at Benares on my return journey. While at Benares we had a very heavy storm of hail and wind, and while my "baboo" was wending his way towards the native part of the city in a homely vehicle called a "Heckler," also called "Jingling Johnny" from the noise it makes, he got caught in the storm, and, with the "Heckler," was blown into a ditch; so he got a good shaking and a ducking.

Lucknow.

City of Palaces. When I say that Lucknow is as full of palaces as an egg is full of meat, I write according to my conscience. The Khaisen Bagh (pronounced barg—bagh means garden) is a very large garden with marble summer-houses, fountains, &c. The garden is walled in on all sides by a fine palace, which was built by the late King of Oude as his harem. Numbers of minarets and domes, all finely carved and gilt, but somewhat shabby now, on account of the purposes for which they are used. The "Hoseinbad Immambara" is a sort of pleasure garden; if it could be carried away and placed in the suburbs of London, a fortune might be realised. The garden has buildings on four sides; on one side is a miniature representation of the "Tarj at Agra"; on another the tomb of one of the Lucknow kings. The tomb is very magnificent. There is a pair of candelabra standing ten feet high, made of Bohemian glass, which cost £1,500; no end of gold and silver poles; a little temple made of gold, studded with diamonds. The inside of the king's tomb puts one in mind of a bazaar. The "Residency" reminds me of Furness Abbey, though some of the rooms are in pretty good condition. The cemetery is within fifty yards of the ruins, and the whole is enclosed in a very large park and kept in excellent order.

My Baboo in Trouble.

The morning after I arrived at Lucknow, before I was up, a letter (*chitthi*) was brought to me from my baboo. It ran thus: "I am laid up with cholera and fever. I shall seek medical assistance and return at once to Calcutta." This pulled me out of the curtains rather earlier than I intended. I at once set out to see him, went up into his "den," felt his pulse, &c., and decided he had better take his bath at once and go out and look for business. This was evidently the right treatment, as he came to me an hour or two afterwards as "bright as a bee." Two or three days passed without my doing any business, when my baboo complained again. I gave him some more of my "patent medicine," which is chiefly composed of plain language, and I told him I was determined to stop, so he had better go and look for some business. He said he could get nothing to eat and nothing to drink. When I told him he looked pretty fat considering, he answered with a sickly smile that the butter was mixed with ghee, the milk was a mixture of buffalos' and goats', and the rice was not good enough. This was a Mahommedan city, and he was a mild Hindoo. I was moved to tears (crocodile's). There was a Rajah staying in the city whom I knew, so I took him there and told the tale of woe. They satisfied his wants and he was as perfectly happy (for the time). But the next morning, when I was in the billiard-room, he appeared with a blanket over his head, just the tip of his nose sticking out, on which he had put a little chalk. He sat himself down on his heels (native fashion) and rocked himself backwards and forwards He had called to tell me "he was going back to Calcutta" (very polite of him). After a great deal of argument I made him consent to his friend stopping with me as my servant, and to be on the right side made him give me the return half of his friend's ticket. I heard afterwards that the baboo was home sick and timid at being away so long.

An Unpleasant Incident.

The most interesting building alongside the River Ganges is the Minarets. It is a temple with two very high columns, nearly as high as the Monument. You can go to the top and have a good view. I got out of the boat here, and proceeded up a great many steps leading up from the river to the foot of the Minarets, when there was a thud, and about half-a-dozen yards from where I stood was a man lying. He had fallen from the top Of course, he was quite dead. He had been bathing with a friend in the river, and then both went to the top of the Minarets. Whether he jumped, fell, or was pushed over I do not know; but his friend (?) was handed over to the police. I did not venture that morning, but did go eventually. I did not stand, as the wind nearly blew my coat off, but contented myself with sitting on the top. I did not feel comfortable, so soon came down. There was no parapet. It was very dangerous.

Gretta.

### THE CHILDREN'S CORNER.

PRIZES.
FOR MOST CORRECT ANSWERS TO PUZZLES.
This Commenced October 1st, 1887.
One Pound a Month.
A PRIZE OF THREE GUINEAS

to the Competitor who shall have sent in the largest number of correct or best answers during the twelve months.

### Puzzles—Second Week.

No. 5 Buried Rivers.—1. A happy New Year to you. 2. The flowers were sent with a message from a friend. 3. My house stands high upon the cliff. 4. Friends ever, never enemies.

No 6. Charade:—

I myself am my *first*,

But my *second*, alas!

Will soon make my *whole*

To my successor pass.

No. 7. Biographical Acrostic.—Initials will give you the name of a poet, finals where he was born. 1. A pattern. 2. A plant and a dye. 3. Something useful on a dark night. 4. Sleepy. 5. A perfumed oil. 6. A system of signs.

No. 8. What vegetable threatens to stifle the Royal Academy?

### Results of Fourth Week Puzzles.

No. 13. Riddle-me-ree.—Crown.

No. 14. Arithmetical Puzzle.

The two prices are $\frac{1}{4}$d. and $1\frac{1}{2}$d each, or any multiple of them.

The *first* sold 49 at $\frac{1}{4}$d. and 1 at $1\frac{1}{2}$d. = $13\frac{3}{4}$ = 1s. $1\frac{3}{4}$d.

The *second* sold 25 at $\frac{1}{4}$d. and 5 at $1\frac{1}{2}$d. = $13\frac{3}{4}$ = 1s. $1\frac{3}{4}$d.

The *third* sold 1 at $\frac{1}{4}$d. and 9 at $1\frac{1}{2}$d. = $13\frac{3}{4}$ = 1s. $1\frac{3}{4}$d..

∴ Each received 1s. $1\frac{3}{4}$d.

No. 15. Enigma.—A dress coat.

No. 16. Geographical Acrostic.

S evre S
E lb A
V oi L
E pso M
R io Negr O
N ewhave N

*All right*—P. S., Plummy Fluff, Jumps, A. C. K., Druce. *Three right*—A. G. S., McCulloch, Scrooge, Robinson, Excelsior, Gem. *Two right*—Judy, Toad, Happy Eliza. *One right*—Spider.

### Results of Fourth Monthly Competition.

The highest number of marks (15) for the past month has been gained by P. S., Druce, Plummy Fluff, Scrooge, and A. C. K. The two latter competitors, however, are disqualified, having already received prizes since this competition commenced in October last. We divide the prize equally between P. S. (Master P. G. Silley, 40, Warwick Gardens, W.), Druce (Master G. D. Piggott, 1, Victoria Terrace, Worthing), and Plummy Fluff (Miss Helen More, 12, Kensington Gardens Terrace, W.), and the sum of 6s. 8d. will be forwarded to each.

All competitors who send in papers must strictly abide by the following

### Conditions.

I. Every paper sent in must be clearly written, and one side only must be used. All competitors must mark at the head of their papers the number of their competition.

II. Each paper must be signed by the author with his or her real name and address. A *nom de plume* may be added, if the writer does not desire to be referred to by us by his real name. In the case of all prize-winners, however, the real name and address will be published.

III. All communications relating to prize competitions should be addressed to "The Prize Editor, The Hospital, 38, Old Jewry, London, E.C." Competitors are requested not to include in letters, etc., to the Prize Editor communications on matters of other business. All letters must be fully prepaid.

IV. The Prize Editor of "The Hospital" is the sole judge of the competitions, and his decision is in all cases final and without appeal.

V. The Prize Editor reserves the right to publish any paper sent in, whether it receives a prize or not. He does not hold himself responsible for any MSS. sent, nor can he undertake to return rejected competitions.

**Notice to all Correspondents.**—N.B.—All letters referring to this page, which do not arrive at 38, Old Jewry, Cheapside, London, E.C., by the *first post on Thursdays*, and are not addressed PRIZE EDITOR, will in future be disqualified and disregarded.

No one will be permitted to win the Monthly or Quarterly Prizes twice in any one year, the annual prizes being offered especially to prevent this.

